# Preconception Care in Low- and Middle-Income Countries: New Opportunities and a New Metric

**DOI:** 10.1371/journal.pmed.1001507

**Published:** 2013-09-03

**Authors:** Cheryl T. Young, Marcelo L. Urquia, Joel G. Ray

**Affiliations:** 1 Keenan Research Centre, Li Ka Shing Knowledge Institute, St. Michael's Hospital, Toronto, Ontario; 2 Department of Medicine and Obstetrics and Gynaecology, University of Toronto, Toronto, Ontario

## Abstract

Joel Ray and colleagues discuss the challenges and opportunities for improving pre-conception care for women in developing countries.

*Please see later in the article for the Editors' Summary*

**Linked Guidelines and Guidance Article**

This Perspective discusses the following new study published in *PLOS Medicine*:

Dean S, Rudan I, Althabe F, Webb Girard A, Howson C, et al. (2013) Setting Research Priorities for Preconception Care in Low- and Middle-Income Countries: Aiming to Reduce Maternal and Child Mortality and Morbidity. PLoS Med 10(9): e1001508. doi:10.1371/journal.pmed.1001508

Sohni Dean and colleagues report their CHNRI exercise that developed health research priorities for effective pre-conception care in low- and middle-income countries.

## Why We Need Preconception Care

Globally, low- and middle-income countries (LMICs) carry a disproportionately heavy burden of maternal and neonatal mortality and morbidity [1]. Too many women haemorrhage to death peripartum, develop acute stroke, renal failure, or pulmonary edema from uncontrolled hypertension, or are affected by severe sepsis, including after unsafe abortion [2]. And when bad things happen to a pregnant woman, the same is likely to be true for her fetus or newborn [3], which can include neonatal sepsis or preterm entry into a world that lacks the facilities to deal with a baby's immature organ system [4]. Indeed, among adolescent mothers and women who deliver in rural or sprawling peri-urban areas, the flagrant persistence of inequitable health outcomes has beckoned experts to generate priorities for action [2], yet preconception care has somehow been neglected. Until now.

Thanks to Sohni Dean and colleagues and reported in this week's *PLOS Medicine*, preconception care has found a place in the continuum of care aimed at improving maternal, newborn, and child health in LMICs [5]. They have created and ranked an extensive list of maternal and obstetrical delivery risk factors, clarified which of those appear amenable to preconception care interventions, and packaged them according to expert-defined criteria like “answerability” and “effectiveness.” The work that went into this is extensive, and we recommend reading their paper more than once.

## Preconception Period—Delaying Conception in Young Women

A pregnancy can be viewed as wanted and planned, wanted but unplanned, unwanted but planned, or unwanted and unplanned. An unwanted and unplanned pregnancy has the highest chance of resulting in poorer maternal or neonatal outcomes [6], yet as a woman's life situation changes, so might her perspective on a future pregnancy. A broad preconception programme can accommodate to this changing reality. By defining the “preconception period” as a minimum of one year prior to the initiation of any unprotected sexual intercourse among adolescents and women of reproductive age, Dean and colleagues advance the discourse on the prioritization of preconception care interventions [7]. Specifically, they point out that pregnancy may arise from unprotected (non-contraceptive) sexual intercourse, including among young women who may not yet have the skills to negotiate decision making regarding their own health. It not only follows that young women need to be taught these skills in late elementary school [8] and thereafter, but also that they be shielded from sexual coercion—whether of a religious, familial, political, or economic origin. Once a woman is ready, the other aspects of an existing preconception care programme, such as micronutrition, anemia prevention, environmental smoke reduction, or HIV prophylaxis can take effect.

It also follows that education in pre-adolescent and adolescent females is a prerequisite for achieving optimal maternal, newborn, and child health. Although mentioned only briefly in their Discussion, Dean and colleagues did not include women's literacy as a necessary means to improving the uptake of preconception care. Literate women are better able to access health resources, understand counselling, and make informed decisions about health planning, subsequently enhancing maternal and child outcomes [8]. Across India, one study suggests that maternal education and literacy has grown to become a more powerful indicator of child malnutrition and survival than poverty [9]. Worldwide, pregnancy complications are the leading cause of death among women aged 15 to 29 years of age, with more deaths in this age group than in any other [2],[3]. Accordingly, interventions that delay pregnancy—such as investments in pre-adolescent and adolescent women's literacy and formal education—may be highly effective in reducing maternal mortality.

## Who Might Best Deliver Preconception Counselling?

A successful preconception care initiative depends not only on a woman having basic literacy skills to comprehend the message, but also on the right messenger. Current initiatives already aim to have a female skilled birth attendant at delivery in LMICs. Not only are they trusted and accessible, but their female character helps circumvent the complexities created by patriarchal cultures in various LMICs. The gender of the skilled birth attendant is a key determinant of health-seeking behaviours of mothers and mothers-to-be [10], wherein females are able to effectively provide sensitive information about how to avoid a pregnancy to the woman not yet prepared for motherhood, or the optimization of her health to the woman who is.

## Measuring the Efficacy of Preconception Care: Age-Specific Maternal Mortality Rate

To assess the impact of a preconception care programme or specific intervention on maternal mortality, one might traditionally use the Maternal Mortality Ratio (MM Ratio)—the number of deaths during or within 42 days after pregnancy, per 100,000 livebirths, wherein the maternal death is from any cause related to or aggravated by the pregnancy (“MM Ratio 1”) (File S1) [11]. However, since some women may die in pregnancy without giving birth, or after an unsafe abortion, the denominator for the MM Ratio really needs to be per 100,000 pregnancies—whether a livebirth, stillbirth, or an abortion—the “MM Ratio 2.” Yet, in the evaluation of a preconception care programme, one must further consider all women capable of becoming pregnant. For that, the right denominator need include all women aged 10–49 years, or those within a specific high-risk age group who might be targeted by a preconception care intervention that aims to delay conception, such as females aged 10–14 years or 15–19 years. Hence, we emphasize that the Maternal Mortality Rate (MM Rate) be used, which describes the number of pregnancy-related deaths per 100,000 women of reproductive age, irrespective of whether a woman is currently pregnant or not (File S1) [11]. Since some preconception care strategies might aim to shift the age of first pregnancy to a higher age group, it makes sense to consider the MM Rate also by age groups, which we call the Age-Specific Maternal Mortality Rate (ASMM Rate):
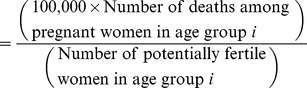
The ASMM Rate can be calculated by five-year age groups for women within the age range of reproductive capacity, and can be used to compare maternal deaths at different age groups, maternal deaths at different age groups over time, or maternal deaths across geographical regions.

The Lifetime Risk of Maternal Death is the percent probability that a hypothetical woman will die in pregnancy or the puerperium across all her childbearing years, calculated as follows:
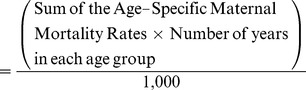


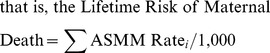
As an example (File S1), two countries have the same number of women of reproductive age in each age group. Although, compared to Country X, Country Y has both half the number of pregnancies and half the number of maternal deaths, their MM Ratios are the same within each age group. Only by also considering their respective ASMM Rate, such as among those aged 10–14 years (30 vs. 15 per 100,000), do we realize that Country X has a higher burden of its young women dying than Country Y. And a hypothetical woman in Country X has a 0.61% Lifetime Risk of Maternal Death in pregnancy or the puerperium, while a woman in Country Y has a 0.31% chance.

Interventions that reduce the total number of pregnancies would eliminate the deaths that would have otherwise occurred due to these pregnancies. The ASMM Rate is particularly useful because it accounts for the lives saved through the postponement or foregoing of pregnancy, while the MM Ratio does not. Moreover, one can extend this approach to severe maternal morbidity measures.

## Supporting Information

File S1 An example comparing maternal mortality using the Maternal Mortality Ratio vs. the Maternal Mortality Rate.
(XLS)

## Abbreviations

ASMM Rate: Age-Specific Maternal Mortality Rate
LMICs: low- and middle-income countries
MM Rate: Maternal Mortality Rate
MM Ratio: Maternal Mortality Ratio
